# Evaluation of the in vivo and in vitro interleukin-12 p40 and p35 subunit response in yellowtail (*Seriola quinqueradiata*) to heat-killed *Lactobacillus plantarum* strain L-137 (HK L-137) supplementation, and immersion challenge with *Lactococcus garvieae*

**DOI:** 10.1016/j.fsirep.2023.100095

**Published:** 2023-04-27

**Authors:** Haruhisa Fukada, Ayaka Senzui, Keisuke Kimoto, Kumiko Tsuru, Yoshikazu Kiyabu

**Affiliations:** a Faculty of Agriculture and Marine Science, Kochi University, 200 Monobe, Nankoku, Kochi 783-8502, Japan; b Bioresource Production Science, The United Graduate School of Agricultural Science, Ehime University, 3-5-7, Tarumi, Matsuyama, Ehime 790-8566, Japan; c Fisheries Research Division, Oita Prefectural Agriculture, Forestry and Fisheries Research Center, Oita 879-2602, Japan

**Keywords:** Interleukin-12 lactobacillus plantarum strain L-137, Lactococcus garvieae, Yellowtail

## Abstract

Dietary supplementation of immunostimulants might be effective to reduce the economic losses due to infectious diseases and the use of antibiotics in aquaculture. To investigate the immune response of interleukin-12 (IL-12) in yellowtail *Seriola quinqueradiata* to heat-killed *Lactobacillus plantarum* strain L-137 (HK L-137), we performed a leukocyte culture, feeding trial with diets containing L-137 and an immersion challenge with *Lactococcus garvieae*. IL-12 (IL-12p70) is a heterodimeric cytokine composed of IL-12p35 and IL-12p40 subunits. In the yellowtail-leukocyte culture, HK L-137 treatment stimulated the mRNA expression of one IL12p35 subunit (*p35a*) and all IL12p40 subunits (*p40*a, *p40b*, and *p40c*) in a dose-dependent manner. Furthermore, mRNA expression of type-I helper (Th-1) cytokine (tumor necrosis factor α, TNF-α, and interferon γ, IFN-γ) was also stimulated by HK L-137. After 6 weeks of feeding yellowtails with diets containing 0, 20, and 100 ppm of HK L-137, the mRNA expression of *p35a* and *p40b* in the spleen leukocytes increased with the dietary concentration of HK L-137, and that of *p40b, p40c*, and *ifng* in the head kidney leukocytes were the highest in the 20 ppm HK L-137 group. Survival rates in the 20 ppm HK L-137 group after immersion challenge with *L. garvieae* were significantly higher than the control (0 ppm of HK L-137). The 100 ppm HK L-137 group did not significantly suppress mortality. HK L-137 showed immunostimulant activity by increasing the expression of *il-12, tnfa*, and *ifng* mRNA in both in vitro and in vivo tests in yellowtail. Our results suggest that dietary supplementation with 20 ppm HK L-137 is the most efficient dose for improving immunity in yellowtail. Furthermore, a high dose of HK L-137 and/or long-term feeding of a diet containing HK L-137 might suppress the immune response, which probably decreases the survival rate of fish. To maintain a high immune response in yellowtail, the optimal dietary concentration of HK L-137 and/or feeding regime should be investigated further.

## 1 Introduction

Aquaculture has become an important industry for meeting global food demand. In 2018, aquaculture finfish production reached 54.2 million tons and contributed to 43.0% of the global finfish fish production [1]. Stable and sustainable production is an important issue for successful aquaculture; however, aquaculture production involves certain risks such as disease, red tide, environmental change, and natural disasters. Fish disease results in the massive loss of cultured fish [2]. Although vaccines for some diseases are currently available and approved for use in some countries, including Japan, it is more common to apply antibiotics and chemotherapy for treatment against pathogenic bacteria [3]. However, the use of antibiotics and chemotherapy may cause drug resistance in both animals and consumers of the fish as well as serious environmental hazards, thus affecting human health directly [2] or indirectly [4]. Furthermore, negative effects such as tissue accumulation, immunosuppression, development of antibiotic-resistant bacteria, and/or destruction of environmental microbiota have also been reported [5].

Probiotics have been used as alternatives to antibiotic use in aquaculture. Probiotics were defined as, “a live microbial feed supplement which beneficially affects the host animal by improving its intestinal balance.” A recent definition postulates that probiotics are “live microorganisms, which confer a health effect on the host when consumed in adequate amounts” [6]. In aquaculture, probiotics improve the growth performance, immune response, and disease resistance of fish [5,7]. Several types of bacteria (*Aeromonas* spp., *Bacillus subtilis, Bacteridaceae, Clostridium* spp., *Lactobacillus plantarum*, and *Staphylococcus* sp.) have been used as probiotics in aquaculture [5]. However, live microbes potentially cause safety concerns in open aquatic environments; therefore, inactivated probiotics might be safer for use in aquaculture [8]. Heat-killed bacteria have been shown to induce a better antibody response when the efficacy of formalinized bacterins and heat-killed *Vibrio vulnificus* bacterins was compared in the olive flounder *Paralichthys olivaceus* [9]. This suggests that heat-killed bacteria can also have positive effects on the immune system of fish.

The probiotic *L. plantarum* promoted growth and enhanced immunity and resistance against *Streptococcus* sp. and an iridovirus in orange-spotted grouper *Epinephelus coioides* [10] Dietary supplementation with *L. plantarum* improved the growth, antioxidant, immune responses, and tolerance of Nile tilapia *Oreochromis niloticus* to *Aeromonas sobria* infection [11]. The L-137 strain of *L. plantarum* is isolated from a fermented fish and rice dish from the Philippines called ‘burong isda’ (i.e., fermented fish) [12]. A heat-killed form of L-137 (HK L-137) is now available commercially. Heat treatment was effective for the immunomodulatory activity of *L. plantarum* L-137 compared to the unheated one in the mouse model [13]. In aquaculture, dietary supplementation of HK L-137 indicated positive effects, including growth performance, digestibility, antioxidative capacity, intestinal integrity stress resistance, and immune response in greater amberjack *Seriola dumerilii* juveniles [14], red sea bream *Pagrus major* [8,15,16], carp *Cyprinus carpio* [17], bighead catfish *Clarias macrocephalus* [18], Nile tilapia [19], and black sea bream *Acanthopagrus schlegelii* fingerlings [20]. Commercially available HK L-137, which is expected to have a stable immune effect compared to probiotics, is useful for various fish diseases, if it has tolerance against various pathogens. However, little research has been conducted to investigate the tolerance of HK L-137 against pathogens, or the immunomodulation of immune-related cytokines by HK L-137.

One function of the HK L-137 is that it is a potent inducer of interleukin-12 (IL-12), and this effect has previously been shown in mice in both in vitro and in vivo studies [21]. IL-12 is a pro-inflammatory cytokine with pleiotropic effects on both the innate and adaptive immune responses. IL-12, consisting of the alpha chain (p35) and beta chain (p40), acts as a heterodimeric IL-12p70, which is mainly expressed in monocytes, macrophages, and dendritic cells. Activated type 1 helper (Th1) cells lead to the priming of macrophages and cytotoxic T-cells via the production of Th1-type cytokines, such as IFN-γ and tumor necrosis factor (TNF) -α. In humans, oral ingestion of HK L-137 increases the Th1-type of immune response (IFN-γ) via IL-12 production [22].

To date, teleost IL-12 genes have been identified in various fishes [23], [24], [25], [26], [27], [28], [29] including two *Seriola* species (greater amberjack and yellowtail) [30]; which include two types of IL-12p35 (*p35a* and *p35b*) and three types of IL-12p40 (*p40*a, *p40b*, and *p40c*). The effect of fish IL-12 on the induction of TNF-α and IFN-γ expression was similar to that in mammals [27,[31], [32], [33], [34]]. However, the regulation of IL-12 expression by HK L-137 has not been fully investigated in fish.

Yellowtail is one of the most important species in Japanese aquaculture and has the highest production in the finfish aquaculture industry in Japan (MAFF: Sea Fishery Production Statistics Survey, https://www.maff.go.jp/j/tokei/kouhyou/kaimen_gyosei/index.html). Various kinds of fish are cultured in Japan, and diverse infectious diseases have been reported in a variety of species. *Lactococcus garvieae* is a harmful pathogenic bacterium in yellowtail aquaculture. Fish infected with *L. garvieae* show high mortality [35]. Between 2016 and 2020, *L. garvieae* caused the greatest of damage in yellowtail aquaculture, which occupied more than 40% of the total amount of damage (https://www.maff.go.jp/j/syouan/suisan/suisan_yobo/disease/attach/pdf/gyobyou_higai_jyoukyou-4.pdf). To reduce the economic losses due to infectious diseases and the use of antibiotics, dietary inclusion of immunostimulants, including HK L-137, might be effective in the aquaculture of different fish species, including yellowtail. In this study, immunomodulatory function of HK L-137 on all subunits of IL-12 was investigated in yellowtail, and bacterial challenge with *L. garvieae* was performed after fish were fed diets containing HK L-137.

## 2 Materials and methods

### 2.1 Preparation of HK L-137

A commercial preparation of heat-killed *L. plantarum* (HK L-137) was obtained from House Wellness Foods Corp. (Itami, Japan), containing 20% heat-killed *L. plantarum* and 80% dextrin on a dry-weight basis [22].

### 2.2 In vitro expression analysis of IL-12 genes in response to HK L-137

Juvenile yellowtail (approximately 200 fish), purchased from Yamasaki Giken (Susaki, Kochi, Japan), were grown at an aquaculture station (Konan, Kochi, Japan) in an indoor 30-ton tank supplied with a continuous flow of filtered seawater. The fish were fed a commercial pellet diet (Hamachi EP; Marubeni Nisshin Feed Co., Ltd., Tokyo, Japan). Yellowtails (*n* = 5, average weight = approximately 1 kg) were collected from the tank. Temperature and photoperiod on the day were 17.0 °C and 11 L:13 D, respectively. Fish were euthanized with an overdose of 2-phenoxyethanol (0.5 mL/L) (Nacalai Tesque Inc., Kyoto, Japan) dissolved in seawater, and blood was collected from each fish. Blood samples were collected from the caudal vessel using a heparinized syringe. Blood was suspended in two volumes of RPMI-1640 containing 5% fetal bovine serum (FBS) and mixed. The diluted blood sample was added to an equal volume of lymphocyte separation solution (density = 1.077 g/mL) (Nacalai Tesque Inc.) and centrifuged at 400 *g* for 30 min at 4 °C. The leukocytes were collected on the surface layer of the lymphocyte separation solution and washed two times with RPMI-1640 containing 5% FBS at 1000 *g* for 10 min at 4 °C. The culture of leukocytes was performed using the modified method of Mori et al. [36]. The isolated leukocytes (1.25 × 10^6^ cells) were harvested in each well of 24-well plates with 1 mL of RPMI-1640 containing 5% FBS overnight. The next day, the culture medium was removed, and then the adherent cells were incubated with 1 mL of RPMI-1640 containing 5% FBS containing various doses of L-137 (0, 20, 100, and 500 ng/mL, 5, 50, and 500 µg/mL, and 5 mg/mL; *n* *=* 6/dose). Incubation times of 3 and 9 h at 25 °C were chosen based on our preliminary test, in which 3, 6, 9, and 24 h incubation times were tested (data not shown). The culture medium was removed after the termination of incubation, and 1 mL of RNAisoPlus (Takara Bio Inc. Shiga, Japan) was added to the wells, and then leukocytes were collected for subsequent real-time quantitative PCR (RT-qPCR). At the end of culture, cell viability was confirmed using 0.4% Trypan blue solution.

### 2.3 Immersion challenge with *L. garvieae*

#### 2.3.1 Fish

Yellowtails used in this study were collected from the coast of Oita Prefecture, Japan, and reared in a 3 × 3 × 3 m sea cage at the Fisheries Research Division, Oita Prefectural Agriculture, Forestry and Fisheries Research Center (Saiki, Oita, Japan) using a commercial extruded pellet (EP) diet (Hamachi EP; Marubeni Nisshin Feed Co., Ltd.). Yellowtails (mean initial weight of 63.3 g) were randomly distributed among three sea cages (3 × 3 × 3 m) with 200 fish in each cage (one cage for one dietary group). All cages were placed in the same small bay and were adjacent to each other. Three experimental diets were prepared using a commercial diet for yellowtail as the basal diet (Feed One Co. Ltd., Kanagawa, Japan) with the addition of 0 (control), 20, and 100 ppm of HK L-137 (House Wellness Foods Corp., Hyogo, Japan) in the diet. The dietary concentration of HK L-137 was determined from previous studies. Twenty parts per million HK L-137 was effective in the survival rate of bacteria challenge in bighead catfish [18] and tilapia [19]. One hundred ppm HK L-137 in low fish meal diet indicated the high growth performance and stress resistance in greater amberjack juveniles [14]. Fish were fed on each of the three experimental diets for 6 weeks, from August 20 to October 5. Water temperature during the feeding trial ranged from 22.3 to 26.3 °C. Dissolved oxygen was >5.0 mg/L during the trial in all cages. On the day after the last feeding, 10 fish were taken randomly from each cage and euthanized with an overdose of 2-phenoxyethanol (0.5 mL/L) (Nacalai Tesque Inc.) to measure the body weight and collect the head kidney, and spleen. The remaining fish were used for the subsequent immersion challenge.

The collected organs were pressed through a cell strainer (100 µm mesh, Corning Inc., Fairport, NY, USA) and suspended in RPMI-1640 containing 5% FBS. The suspension was applied to a lymphocyte separation solution (density = 1.077 g/mL) (Nacalai Tesque Inc.) and centrifuged at 400 *g* for 30 min at 4 °C. The leukocytes were collected from the surface layer of the lymphocyte separation solution and washed two times with RPMI-1640 containing 5% FBS at 1000 *g* for 10 min at 4 °C. After washing, the collected leukocytes were immersed in RNAlater solution (Thermo Fisher Scientific, Waltham, MA, USA) and stored at −80 °C until mRNA expression was measured.

#### 2.3.2 Immersion challenge

We used *L. garvieae* (strain number 091,268, identified as type I) for the immersion challenge in this study. The strain was originally isolated from a yellowtail brain during an outbreak at a culture farm in Oita Prefecture, Japan, and stored at −80 °C. After thawing, the bacterium was inoculated into 7 mL brain heart infusion broth (Becton, Dickinson and Company, Franklin Lakes, NJ, USA) supplemented with 1.5% NaCl (BHI) and precultured at 25 °C with shaking at 100 rpm for 16 h. Next, 0.75 mL of the suspension was inoculated into 300 mL of BHI broth, incubated at 25 °C with shaking at 100 rpm until middle-to-late logarithmic culture phase for 15‒17 h.

Fish (*n* *=* 22 or 23 from each dietary group) fed with three experimental diets for 6 weeks were taken from each of the three dietary groups (0 ppm as control, 20 ppm and 100 ppm HK L-137 groups). Fish were anesthetized with 2-phenoxyethanol (0.1 mL/L) (Nacalai Tesque Inc.) and then marked individually with a visual implant elastomer (Northwest Marine Technology, Inc., Anacortes, WA, USA). The fish were then immersed in a 100-L capacity tank containing *L. garvieae* at a 5.8 × 10^5^ CFU/mL concentration for 10 min. After the challenge, the fish were held in a single 2000 L capacity tank with running seawater at a water temperature of 22–23 °C and a dissolved oxygen saturation rate of 85–100% and reared without feeding for 22 days (October 5‒26). On the last day of the immersion challenge, all surviving fish were examined for *L. garvieae* infection by reisolating the bacterium from the brain and kidney on trypticase soy agar plates (Becton, Dickinson and Company).

### 2.4 RT-qPCR for IL-12, TNF-α, and IFN-γ

Total RNA from leukocytes after in vivo and in vitro treatment with HK L-137 was extracted using RNAisoPlus (Takara Bio Inc.) according to the manufacturer's instructions. RNA purity was verified by the optical density (OD) absorption ratio, and samples with OD 260 nm/OD 280 nm <1.8 were excluded from analysis. Reverse transcription was performed with ReverTra Ace qPCR RT Master Mix with gDNA Remover (Toyobo Co., Ltd., Umeda, Osaka, Japan) using 3 µL RNA solution (300 ng/µL). The primer and proves sequences used for RT-qPCR are listed in Table 1. The NCBI gene accession numbers for each gene are **LC146391** for IL-12 p35a, **LC146392** for IL-12 p35b, **LC146393** for IL-12 p40a, **LC146394** for IL-12 p40b, **LC146395** for IL-12 p40c, **LC545587** for IFNγ, and **LC010967** for TNFα. Primers and probes for RT-qPCR were designed using the primer express program (Thermo Fisher Scientific). RT-qPCR was performed using GoTaq qPCR Master Mix (Promega, WI, USA) for IL-12 p35a, IL-12 p35b, IL-12 p40a, IL-12 p40b, IL-12 p40c, IFN-γ, and TNF-α. The PCR reaction for the assay (20 µL) contained 10 µL of GoTaq qPCR, 2 µL of first-strand cDNA, 1 µM each of forward and reverse primers, and 0.2 µM of probe. Prior to RT-qPCR, gene expressions of elongation factor 1 alpha (*ef1a*), 18S ribosomal RNA, and β-actin were evaluated in the leucocyte. The GeNorm2 algorithm [37] from the R software package (ctrlGene) was used to compare the expression stabilities of the candidate reference genes statistically. Owing to the highest stability of *ef1a* mRNA, it was selected as the reference gene for RT-qPCR analysis. The *ef1a* mRNA expression levels have been shown to be stable in the head kidney, and spleen of Atlantic salmon *Salmo salar* [38]. Furthermore, the *ef1a* mRNA gene indicated stable expression also in the leucocytes, head kidney, and spleen of yellowtail of this study. Power SYBR Green PCR Master Mix (Thermo Fisher Scientific) was used to amplify the *ef1α* gene. The PCR reaction for the assay (20 µL) contained 10 µL Power SYBR Green PCR Master Mix (Thermo Fisher Scientific), 2 µL of first-strand cDNA (1/100 dilution), and 0.6 µM each of forward and reverse primers. Samples were amplified and detected using the StepOnePlus system (Thermo Fisher Scientific) with the following thermal cycling conditions: 95 °C for 2 min, 95 °C for 15 s, and 60 °C for 1 min (50 cycles). Four serial dilutions of sample cDNA were run to determine the efficiency (E) of PCR, which was calculated from the regression slope of the assay (*E* = 10^(−1/slope)^). Yellowtail mRNA expression of target genes was calculated relative to *ef1a* mRNA using the method described by Pfaffl [39].

**Table 1:** Primer and probe sequences used for the yellowtail RT-qPCR.

Gene	Primer/Probe	Sequence	Efficiency (%)
IL12 p35a	Forward	CAGAGAGCAGATGTGTAAGATGATGA	101.6
LC146391	Reverse	GTAGCCCATAGCTCGGTTGA	
	Probe	[FAM] AGGTTTCCATGTCCGAGCCATCACC [TAM]	
IL12 p35b	Forward	GACCTGCGTCACTACTATAAATTTCTCA	100.8
LC146392	Reverse	GAGGCTGGACAGAACAATTTTGT	
	Probe	[VIC] AGCCCAGCCGGACCCTTCTAAGTTACTT [TAM]	
IL12 p40a	Forward	CCTGGAGCAGTCCCTACTCCTA	97.4
LC146389	Reverse	TTTGCATCCCCCCTTCAG	
	Probe	[FAM] TTCCCCCTCACTTTCCAGATTGCACA [TAM]	
IL12 p40b	Forward	GCCCATACGGAGAGGAACAA	98.9
LC146390	Reverse	TGTAGGACTCGAGGCGAGAGTAG	
	Probe	[FAM] AACGCATCTCCCTCACCGTTTACATACACA [TAM]	
IL12 p40c	Forward	CCCTACGCTGAGGAAACCAA	101.9
LC146393	Reverse	TCCTGAGGATGTTGTGGTTCATT	
	Probe	[FAM] ATGCTTGTACTCACTGCCGAGGCCA [TAM]	
IFN-γ	Forward	CATTACCAGGAGCAGGACATGT	101.0
LC545587	Reverse	CTTTGTTCTGGATGATGAGGTCAT	
	Probe	[FAM] CTGCAGGCCCTCAAACACATCAAGATG [TAM]	
TNF-α	Forward	CCACTACACGCTGAAGCGAAT	106.8
LC010967	Reverse	TCGTAGCTACCTTCTAAATGG	
	Probe	[VIC] AGCAGCAAAGCCAAGGCAGCCA [TAM]	
EF1A*	Forward	GGCCAGATCAATGAGGGTTA	100.2
LC010962	Reverse	ATGGGCTTCTGTGGAATGAG	

⁎ Jirapongpairoj et al., 2015. Accession No. of each gene was noted under the gene name.

### 2.5 Statistical analysis

Gene expression levels in the leukocyte cultures were statistically analyzed using two-way analysis of variance (ANOVA) followed by Sidak's multiple comparison tests. Body weight and gene expression levels after the 6-week feeding trial were statistically analyzed using a one-way ANOVA followed by Tukey's multiple comparison tests, and the log-rank (Mantel-Cox) test was used for survival tests to compare between the control and test groups. Statistical analyses were performed using GraphPad Prism 9 (GraphPad Software, San Diego, CA, USA). Differences between groups were considered statistically significant at *p* <0.05.

### 2.6 Animal ethics

All experiments were performed based on Animal Experiments at Act on Welfare and Management of Animals (Notice of the Ministry of the Environment No. 105 of 1973), Reducing Pain of Laboratory Animals (Notice of the Ministry of the Environment No. 88 of 2006).

## 3 Results

### 3.1 In vitro il12, ifng, and tnfa gene response to HK L-137

**Fig. 1:**
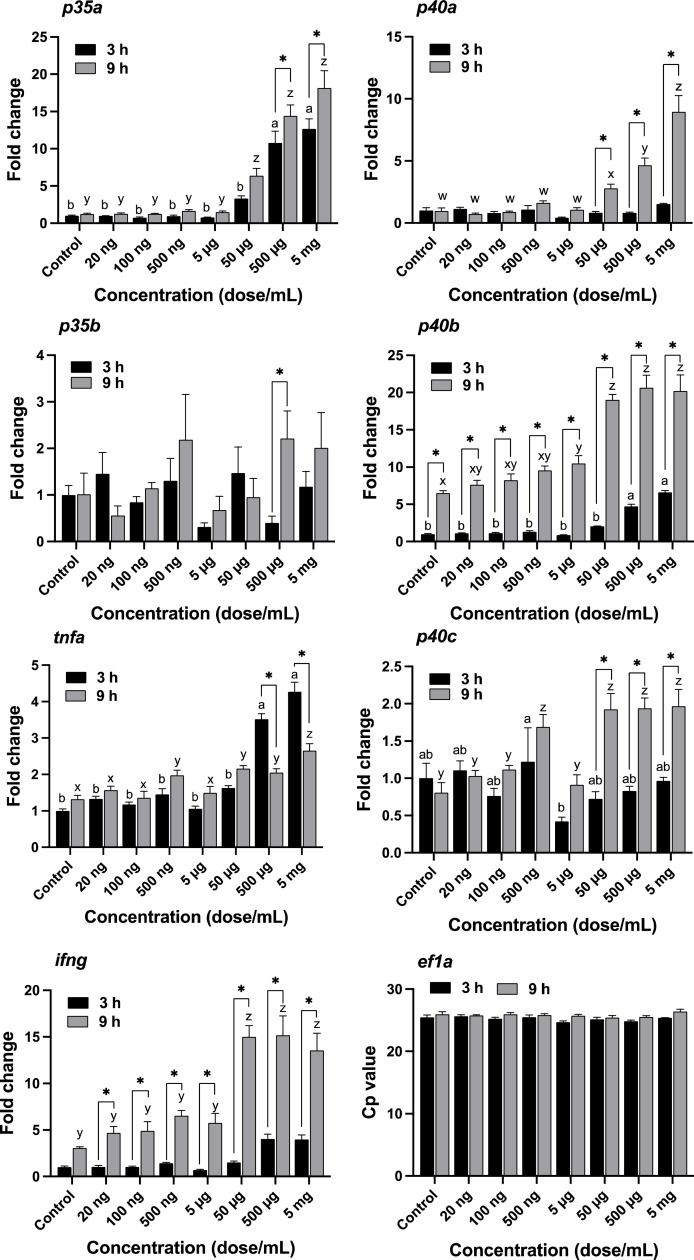
Modulated expression of *p35a, p35b, p40a, p40b, p40c, tnfa, ifng*, and *ef1a* in response to different doses of HK L-137 in the leukocyte culture of yellowtail. mRNA expression of the control (no addition of HK L-137) was used as 1 to normalize the data. Vertical lines represent the standard error of the mean (SEM; *n* = 5–6). Lowercase a and b indicate significant differences at 3 h, and lowercase x and y indicate significant differences at 9 h using two-way ANOVA followed by Tukey's multiple comparison test (*p* <0.05). Asterisks indicate significant differences between 3 and 9 h of incubation time using two-way ANOVA followed by Sidak's multiple comparison test (*p* <0.05).

The expression of *il12, ifng,* and *tnfa* genes in response to HK L-137 under in vitro conditions is shown in Fig. 1.

The average cell viability was 97% in this study. The crossing point (Cp) of *ef1a* mRNA in RT-qPCR did not indicate a significant difference between incubation times (Fig. 1).

The mRNA expression of *p35a* increased in a dose-dependent manner at both 3 and 9 h of incubation. *p35a* mRNA expression was significantly higher after 9 h of incubation than after 3 h of incubation in the 500 µg/mL and 5 mg/mL HK L-137 groups. Expression of *p35b* mRNA did not change with the dose of HK L-137. The only significant difference was observed in the 500 µg/mL treatment between 3 h and 9 h, wherein *p35b* mRNA expression was higher at 9 h than at 3 h.

Expression of *p40a* mRNA did not change after 3 h of incubation. After 9 h of incubation, *p40a* mRNA expression increased in a dose-dependent manner up to 50 µg/mL and was maintained at a high level up to 5 mg/mL. Furthermore, *p40a* mRNA expression was significantly higher after 9 h of incubation than after 3 h of incubation at 50 and 500 µg/mL and 5 mg/mL of HK L-137. Expression of *p40b* mRNA increased in a dose-dependent manner at both 3 and 9 h of incubation. Furthermore, *p40b* mRNA expression was significantly higher after 9 h of incubation than after 3 h of incubation in all doses of HK L-137. The *p40c* mRNA expression was significantly higher in the 500 ng/mL treatment than in the 5 µg/mL at 3 h. After 9 h of incubation, the 500 ng/mL, 50 and 500 µg/mL, and 5 mg/mL treatments showed significantly higher expression than the other dose treatments. Furthermore, *p40c* mRNA expression was significantly higher after 9 h of incubation than after 3 h of incubation at 50 and 500 µg/mL and 5 mg/mL of HK L-137.

Significantly higher expression of *tnfa* was observed in the 500 µg/mL and 5 mg/mL treatment groups compared to the other dose treatments at 3 h. Significantly low levels of *tnfa* were observed in the 500 µg/mL and 5 mg/mL treatments after 9 h of incubation compared to those after 3 h. Expression of *ifng* mRNA did not change with the dose of HK L-137 at 3 h. After 9 h of incubation, the 50 and 500 µg/mL, and 5 mg/mL treatments showed significantly higher *ifng* mRNA expression compared to other dose treatments. Furthermore, significantly higher expression of *ifng* was observed in all dose treatments except control after 9 h of incubation than in those after 3 h.

### 3.2 In vivo il12, ifng, and tnfa gene response to HK L-137

#### 3.2.1 Fish

After 6 weeks of the feeding trial, fish body weight (*n* *=* 10) was measured individually in each cage. The fish in the control group weighed 276.0 ± 8.1 g, whereas those in the 20 and 100 ppm HK L-137 groups weighed 264.7 ± 11.1 g and 274.0 ± 8.8 g, respectively. No significant differences were observed in the body weights of different groups (*p* <0.05).

#### 3.2.2 il12, ifng, and tnfa expression in head kidney leukocytes of yellowtails

**Fig. 2:**
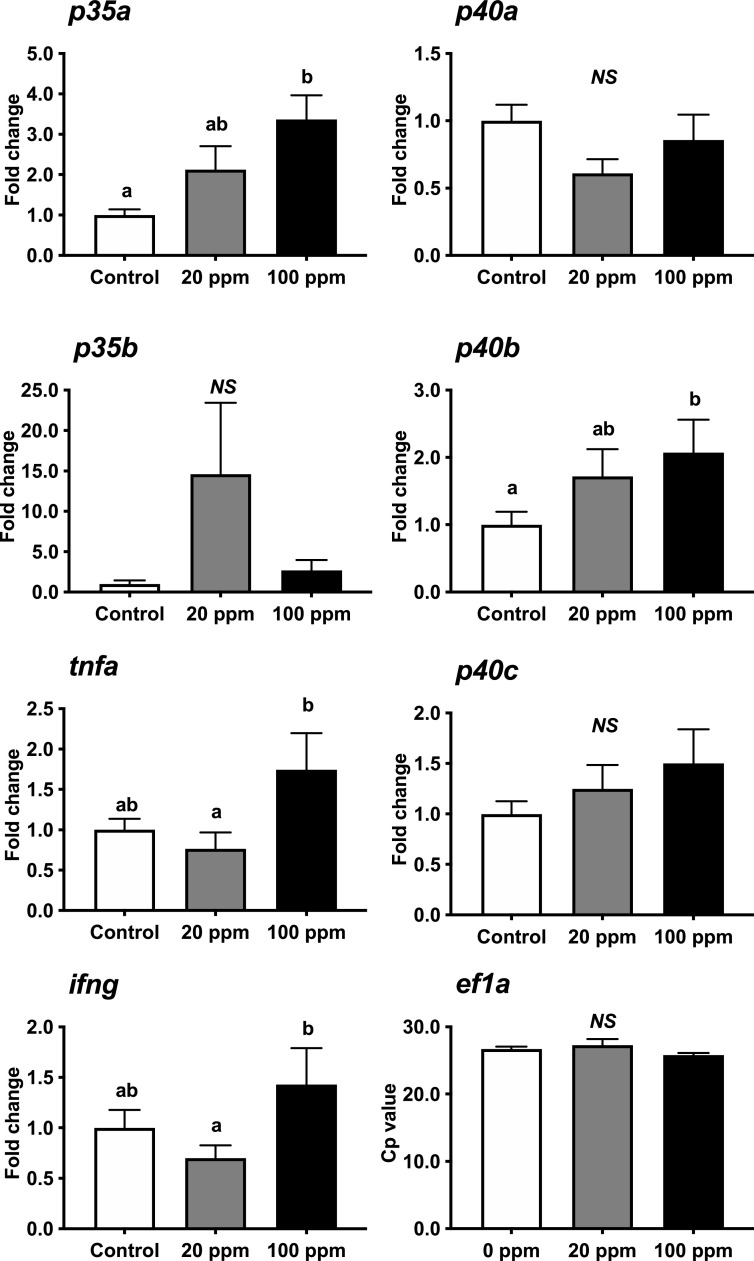
Modulated expression of *p35a, p35b, p40a, p40b, p40c, tnfa, ifng*, and *ef1a* in response to different doses of HK L-137 in the head kidney leukocytes of yellowtail fed with the experimental diets for 6 weeks. mRNA expression of control (no addition of HK L-137) was used as 1 to normalize the data. Vertical lines represent the standard error of the mean (SEM; *n* = 9–10, except *p35b; n* = 5–7). Lowercase letters indicate significant (*p* <0.05) differences among the three dietary groups.

The expression of *il12, ifng,* and *tnfa* genes in the head kidney leukocytes is shown in Fig. 2. The Cp of *ef1a* mRNA in RT-qPCR did not indicate significant difference among dietary groups. In the head kidney leukocytes, the expression of *p35a* and *p35b* mRNA was not significantly affected by the dietary concentrations of HK L-137. The expression levels of *p35b* mRNA were extremely low (almost no amplification) in the 3–5 fish/dietary group. They could not be measured with our RT-qPCR analysis.

The mRNA expression of *p40a, p40b*, and *p40c* indicated a similar trend among the three dietary groups. The highest expression was observed in the 20 ppm HK L-137 group, which was significantly higher than that in the 100 ppm HK L-137 group except for *p40a*.

The expression of *tnfa* mRNA was not significantly affected by the dietary concentration of HK L-137 among the three dietary groups. *ifng* mRNA expression was highest in the 20 ppm HK L-137 group and lowest in the 100 ppm HK L-137 group.

#### 3.2 3. il12, ifng, and tnfa expression in the spleen leukocytes

**Fig. 3:**
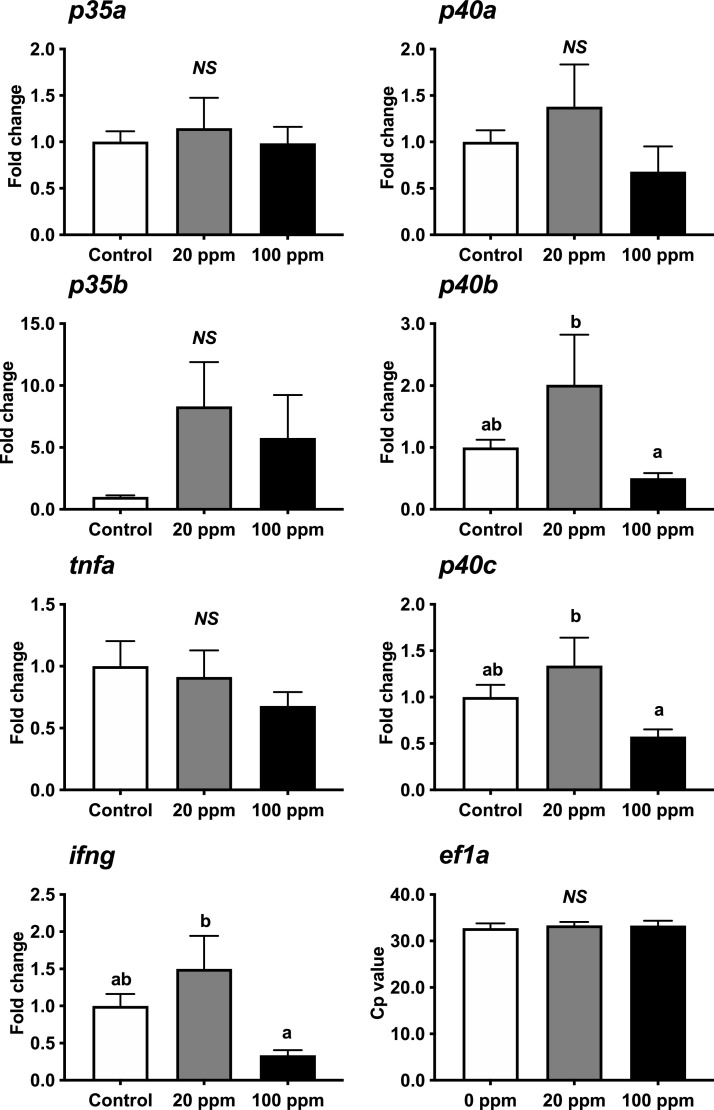
Modulated expression of *p35a, p35b, p40a, p40b, p40c, tnfa, ifng*, and *ef1a* in response to different doses of HK L-137 in the spleen leukocytes of yellowtail fed on the experimental diets for 6 weeks. mRNA expression of control (no addition of HK L-137) was used as 1 to normalize the data. Vertical lines represent the standard error of the mean (SEM; *n* = 8–10). Lowercase letters indicate significant (*p* <0.05) differences among the three dietary groups.

The expression of *il12, ifng,* and *tnfa* genes in the spleen leukocytes are shown in Fig. 3. The Cp of *ef1a* mRNA in RT-qPCR did not indicate significant differences among dietary groups. In the spleen, *p35a* mRNA expression increased in a concentration-dependent manner in HK L-137-treated cells. Expression of *p35b* mRNA did not change significantly with the dietary HK L-137 concentration. mRNA expression of *p40a* and *p40c* did not change significantly, whereas that of *p40b* increased in a dose-dependent manner with increasing dietary HK L-137 concentrations.

The expression of *tnfa* and *ifng* mRNA was high in the 100 ppm HK L-137 group, which was significantly different from that in the 20 ppm HK L-137 group.

#### 3.2.4 Immersion challenge with L. garvieae

**Fig. 4:**
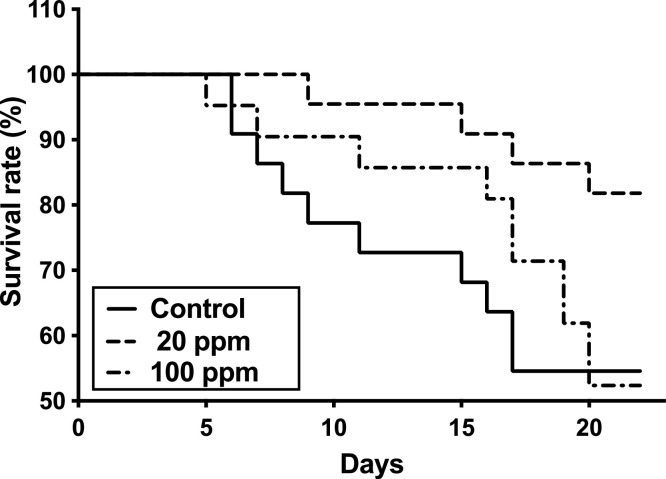
Survival rate of yellowtail fed different concentrations of HK L-137 at 22 day post immersion-challenge with *Lactococcus garvieae.* Control, 0 ppm of HK L-137 in diet; 20 ppm, 20 ppm of HK L-137 in diet; and 100 ppm, 100 ppm of HK L-137 in diet. The log-rank (Mantel–Cox) test was used for survival tests to compare between the control and test groups (*p* <0.05).

The survival rate after 22 days of the immersion challenge is shown in Fig. 4. The number of distinguished fish was 22 for the 0 and 20 ppm groups and 21 for the 100 ppm group. On day 11, the survival rates of the 0, 20, and 100 ppm groups were 72.7%, 95.5%, and 90.5%, respectively. On day 22, the survival rates of the 0, 20, and 100 ppm groups were 54.5%, 81.8%, and 52.4%, respectively. The survival rate in the 20 ppm group was significantly higher than that in the control group (*p* <0.05).

## 4 Discussion

### 4.1 In vitro il12, ifng and tnfa gene response to HK L-137

In aquaculture, dietary supplementation with prebiotics, probiotics, and related substances (c.f. HK L-137) are good options for reducing antibiotic use and loss of fish by disease. The effects of HK L-137 on fish have been confirmed in Nile tilapia [19], bighead catfish [18], red sea bream (*Pagrus major*) [8,15,16,40] and greater amberjack [14]. In these studies, HK L-137 improved the growth performance, immune responses, and stress resistance of the fish. In bighead catfish [18] and Nile tilapia [19], cumulative mortality during the bacterial challenge was suppressed by dietary supplementation with HK L-137. Increased lysozyme activity, resistance to oxidative stress, and mucus amount on exposure to HK L-137 may contribute to a reduced mortality rate. HK L-137 has been reported to induce Th-1 type immune responses in mice [21]. Lipoteichoic acid in HK L-137, a predominant surface glycolipid of gram-positive bacteria, induced IL-12-like LPS in the TPH-1 cell [41]. In fish, recombinant IL-12 plays an important role in immunity in orange-spotted grouper [28] and greater amberjack [30,31]. We first investigated whether HK L-137 induces gene expression of IL-12, TNF-α and IFN-γ in the culture of yellowtail leukocytes.

IL-12 is composed of a heterodimer of p35 and p40 subunits, and the p35 subunit is a member of the IL-6 family, whereas the p40 subunit resembles a soluble cytokine receptor [42]. In mice, a dose-dependent increase in IL-12, TNF-α, and IFN-γ protein levels induced by HK L-137 treatment was observed in vitro. In addition, HK L-137 injection caused a dose-dependent increase in plasma IL-12 levels in mice [21]. The levels of p40 subunit protein in the culture of mouse spleen cells were increased by HK L-137 supplementation in a dose-dependent manner [43]. However, the induction of IL-12, TNF-α, and IFN-γ mRNA expression by HK L-137 has not been extensively studied in fish. In teleosts, a change in the mRNA expression of IL-12 after HK L-137 supplementation was only observed in tilapia, in which IL-12 (only single *p35* subunit) mRNA expression increased in fish fed an experimental diet containing HK L-137 [44]. Some teleosts have two or three types of IL-12p35 and three types of IL-12p40 [23], [24], [25], [26], [27], [28], [29], [30]. The response of each subunit to stimulants has not been fully confirmed in teleosts.

In the leukocyte culture of yellowtail, the expression of *p35a* and *p40a* mRNA indicated an early response to HK L-137 compared to other IL-12 subunits. IL-12 in Atlantic salmon, *p35a1, p35a2*, and *p35b* were confirmed [33]. When LPS was applied to the head kidney leukocyte culture, only *p35a1* expression increased, whereas *p35a2* and *p35b* did not respond. Upon applying poly (I:C), *p35a1* and *p35a2* increased, but *p35b* did not respond. No significant response of *p35b* to stimulants was recorded in previous studies, similar to our results. In greater amberjack, *p35a* responded faster than *p35b* when formalin-killed cells (FKCs) of a pathogen (*Nocardia seriolae*) were added to the culture medium of head kidney leukocytes [30]. In yellowtails, two IL-12p35 units might have distinct functions; furthermore, IL-12p35a might respond to pathogen infection.

For IL-12p40, two IL-12p40 (p40b and p40c) subunits were reported in rainbow trout (*Oncorhynchus mykiss*) [34], and three IL-12p40 (p40a, p40b, and p40c) were confirmed in common carp [24] and grass carp [45]. In rainbow trout, both *p40b* and *p40c* responded similarly when LPS and poly (I:C) were added to the primary culture of head kidney macrophages [34]. Three subunits of *p40* responded to LPS, and the maximum increase in mRNA expression of *p40b* was observed in head kidney leukocytes of grass carp [45] and head kidney macrophages of common carp [24]. Furthermore, the gene expression of three IL-12p40 (*p40b1, p40b2*, and *p40c*) was measured in Atlantic salmon, wherein *p40b1* and *p40b2* were stimulated by LPS and poly (I:C), but *p40c* did not show any response [34]. In greater amberjack (*p40a, p40b,* and *p40c*), *p40a* and *p40b* responded to the FKCs of *N. seriolae* [30]. Furthermore, recombinant IL-12 protein with the combination of both IL-12p35 subunits and p40a/p40c subunits induce Th1-cytokine IFN-γ expression and suppress Th2-cytokine IL-10 mRNA by co-stimulation with FKCs of *N. seriolae,* whereas recombinant IL-12 protein with the combination of both IL-12p35 subunits and p40b subunit only down regulated *IL-10* expression in greater amberjack [30]. IL-10 suppressed IFN-γ production in Th1 cells in mice [46]. In yellowtail, *p40b* showed extremely high amplification by HK L-137. These results suggest that the regulation of the three subunits of *p40* expression was distinct, and that each have different roles when yellowtail is infected by pathogens. However, the increase of *p40b expression* in the control was observed at 9 h incubation. It is likely that *p40b* responded to the medium including FBS. Further studies are needed to evaluate the immunomodulation of HK L-137 on the regulation of Th-1 cytokine including IL-10 (produced in Th-2) and *p40b* expression in yellowtail leucocytes.

As Th-1 cytokines, the gene expression levels of *tnfa* and *ifng* were measured. TNF-*α* and IFN-*γ* have anti-inflammatory and antiviral activities in puffer fish, respectively [47]. In yellowtail, *tnfa* showed a faster response compared to *ifng* after HK L-137 treatment. In greater amberjack, *ifng* mRNA expression was not stimulated with FKCs of *N. seriolae* but was stimulated with recombinant IL-12 protein in the head kidney leukocytes and spleen leukocytes [31]. Expression of *ifng* mRNA in rainbow trout was also stimulated by recombinant IL-12 protein [34]. HK L-137 might stimulate *tnfa* expression directly and *ifng* indirectly via IL-12; therefore, their response times differed. Recent research has indicated that recombinant IL-12 protein also stimulates *tnfa* expression directly in grass carp [45]. HK L-137 may directly stimulate yellowtail *tnfa*, but the production ratio by direct stimulation of IL-12 is probably small. Immunostimulant activity for *il12, ifng*, and *tnfa* cytokine mRNA expression by HK L-137 was observed at 50 µg/mL HK L-137 dose in this study. This dose was similar to that of LPS (25 µg/mL) and poly (I:C) (50 µg/mL) when the induction of IL-12 in the head kidney culture of Atlantic salmon [33] and rainbow trout [34] was investigated. The immunomodulatory activity of HK L-137 was similar to that of LPS and poly (I:C), suggesting that HK L-137 has antimicrobial and antiviral effects in yellowtail.

### 4.2 Immersion challenge

After the feeding trial, the average body weights of 10 fish from each dietary group did not differ significantly. However, because sample number was small, the effects of HK L-137 on growth performance should be confirmed in the future. Relative high-dose HK L-137 supplementation with/without other substances to diets improved the growth and feed efficiency in red sea bream [8,15], greater amberjack juveniles [14], bighead catfish [18], Nile tilapia [19], and black sea bream fingerlings [20]. Therefore, dietary supplementation of HK L-137 has no negative effect on the growth performance of yellowtail.

In our immersion challenge with *L. garvieae*, fish fed a diet containing 20 ppm of HK L-137 showed the highest survival rate. The survival rate in the 100 ppm HK L-137 group was not significantly different from that of the control group at the end of the challenge, although a higher survival rate was observed in the middle of the challenge compared to that in the control group. The dietary concentration of HK L-137 might be important for improving the survival rate in pathogen infection. In tilapia, when diets containing 0, 10, 20, and 50 ppm of HK L-137 were tested, only 20 ppm of the HK L-137 group showed significantly lower cumulative mortality than the 0 ppm HK L-137 group in bacterial challenge using *Streptococcus agalactiae* [19]. Phagocytic and lysozyme activities increased in tilapia that were fed diets containing 20 and 50 ppm of HK L-137, which might result in low cumulative mortality in the 20 ppm HK L-137 group. In bighead catfish, cumulative mortality was significantly lower in fish fed with diets containing 10 ppm of HK L137 than in fish fed with diets containing 0, 20, and 50 ppm of HK L-137 [18]. Fish fed diets containing 20 ppm HK L-137 also showed significantly lower mortality compared to 0 and 50 ppm of HK L137 in the dietary group. The highest lysozyme activity in fish fed a diet containing 20 ppm HK L137 might contribute to low cumulative mortality in bighead catfish. From these reports, the optimal dietary concentration of HK L-137 might exist to elicit immune responses against pathogens. In yellowtails, 20 ppm of HK L-137 in the diet may be optimal; however, this result was evaluated from a single tank and this optimal concentration could vary depending on the feeding period, fish size, and rearing conditions. Also, fish were not fed during this immersion challenge, and fasting may induce physiological and immunological responses in yellowtail. Additional research is required to determine the efficacy and the optimal dietary concentration of HK L-137 in yellowtails.

*il12, ifng,* and *tnfa* genes were measured after 6 weeks of the feeding trial (before the immersion challenge). Because gene expression of *il12* was low, isolation of leukocytes was performed. The following discussion is based on the possibility that gene expression levels may have been affected by this separation. In our study, the response of these genes indicated different expression patterns based on dietary concentrations of HK L-137 and between the head kidney and spleen leukocytes. Different expression patterns between tissues were observed in IL-12p40 (corresponding to *p40c*) of rock bream *Oplegnathus fasciatus*, with peak expression levels at 48 h in the kidney and 24 h in the spleen when infected with *Vibrio alginolyticus* [29]. In yellowtails, the expression of *40b* mRNA, which indicates the highest amplification against HK L-137 in vitro, increased along with the dietary concentration of HK L-137 in the spleen leucocytes but was the highest in the 20 ppm HK L-137 group head kidney leucocytes. A similar trend was also observed for *p35a* and *p40c* in yellowtail. In our leukocyte culture, expression of *p40b, p40c*, and *ifng* reached a plateau from 50 µg/mL of HK L-137, and *tnfa* decreased after 9 h of incubation compared to that at 3 h of incubation. Furthermore, the expression of *p35b* in the head kidney leukocytes was not detectable in some fish with our method. Therefore, negative feedback might have occurred by feeding the fish a diet containing high levels of HK L-137 for a long time. In European seabass *Dicentracus labrax*, with *p35* and *p40* genes, *p35* expression was not stimulated by a bacterial infection in the head kidney culture [23]. The hematopoietic nature of the head kidney cells may be different in IL-12 expression compared to that in the spleen [48]. Furthermore, expression of *tnfa* and *ifng* mRNA in 100 ppm of HK L-137 was the highest in the spleen, whereas it was the lowest in the head kidney leukocytes. This might be one of the reasons for the lack of significant suppression of fish mortality in the 100 ppm HK L-137 group and the highest survival rate in the 20 ppm HK L-137 group. Long-term administration of glucan as an immunostimulant in gilthead seabream (*Sparus aurata*), resulted in negative effects on its resistance against *Pasteurellosis* [49]. Rainbow trout fed with high doses of glucans were more susceptible to infection by *Flexibacter columnaris* [50]. High doses and/or long-term administration of immunostimulants might have negative or no effects on immune factors and pathogen infection. Considering the low expression of *p40a, p40b, p40c*, and *ifng* in the head kidney leukocytes in the 100 ppm HK L-137 group, high dose and/or long-term intake of HK L-137 might decrease the expression of *il12, ifng,* and *tnfa* cytokine genes. Under our experimental conditions, 20 ppm of HK L-137 was optimal from IL-12, IFN-γ, and TNF-α mRNA expression in the head kidney leukocytes and for the survival rate. Investigating the optimal dietary concentration of HK L-137 is needed for future experiments. To determine the optimal dietary concentration and/or feeding regime of HK L-137, the analysis of mRNA expression of *il12, ifng,* and *tnfa* mRNA might be useful.

In conclusion, HK L-137 has immunostimulant activity in the expression of *il12, ifng,* and *tnfa* mRNA in an in vitro test of yellowtails. Furthermore, HK L-137 improved the survival rate of bacteria challenged at 20 ppm in the diet by stimulating the mRNA expression of *il12, ifng,* and *tnfa*. However, a high dose of dietary HK L-137 and/or long-term feeding of the diet containing HK L-137 might decrease the immune response, which might result in a decreased survival rate of fish. The optimal dietary concentration of HK L-137 and the feeding regime should be investigated to maintain a high immune response. This makes HK L-137 a useful tool to reduce mortality in aquaculture.

## Funding

This research was partly supported by House Wellness Foods Corp.

## CRediT authorship contribution statement

**Haruhisa Fukada:** Conceptualization, Investigation, Writing – review & editing. **Ayaka Senzui:** Investigation. **Keisuke Kimoto:** Investigation. **Kumiko Tsuru:** Investigation. **Yoshikazu Kiyabu:** Project administration.

## Declaration of Competing Interest

The authors declare the following financial interests/personal relationships which may be considered as potential competing interests:

Haruhisa Fukada reports financial support was provided by House Wellness Foods Corp.

## Data Availability

The data sets generated during and/or analysed during the current study are available from the corresponding author on reasonable request.
